# Knowledge and Skills of Healthcare Providers in Sub-Saharan Africa and Asia before and after Competency-Based Training in Emergency Obstetric and Early Newborn Care

**DOI:** 10.1371/journal.pone.0167270

**Published:** 2016-12-22

**Authors:** Charles A. Ameh, Robert Kerr, Barbara Madaj, Mselenge Mdegela, Terry Kana, Susan Jones, Jaki Lambert, Fiona Dickinson, Sarah White, Nynke van den Broek

**Affiliations:** Centre for Maternal and Newborn Health, Liverpool School of Tropical Medicine, Liverpool, United Kingdom; Centre Hospitalier Universitaire Vaudois, FRANCE

## Abstract

**Background:**

Healthcare provider training in Emergency Obstetric and Newborn Care (EmOC&NC) is a component of 65% of intervention programs aimed at reducing maternal and newborn mortality and morbidity. It is important to evaluate the effectiveness of this.

**Methods:**

We evaluated knowledge and skills among 5,939 healthcare providers before and after 3–5 days ‘skills and drills’ training in emergency obstetric and newborn care (EmOC&NC) conducted in 7 sub-Saharan Africa countries (Ghana, Kenya, Malawi, Nigeria, Sierra Leone, Tanzania, Zimbabwe) and 2 Asian countries (Bangladesh, Pakistan). Standardised assessments using multiple choice questions and objective structured clinical examination (OSCE) were used to measure change in knowledge and skills and the Improvement Ratio (IR) by cadre and by country. Linear regression was performed to identify variables associated with pre-training score and IR.

**Results:**

99.7% of healthcare providers improved their overall score with a median (IQR) increase of 10.0% (5.0% - 15.0%) for knowledge and 28.8% (23.1% - 35.1%) for skill. There were significant improvements in knowledge and skills for each cadre of healthcare provider and for each country (p<0.05). The mean IR was 56% for doctors, 50% for mid-level staff and nurse-midwives and 38% for nursing-aides. A teaching job, previous in-service training, and higher percentage of work-time spent providing maternity care were each associated with a higher pre-training score. Those with more than 11 years of experience in obstetrics had the lowest scores prior to training, with mean IRs 1.4% lower than for those with no more than 2 years of experience. The largest IR was for recognition and management of obstetric haemorrhage (49–70%) and the smallest for recognition and management of obstructed labour and use of the partograph (6–15%).

**Conclusions:**

Short in-service EmOC&NC training was associated with improved knowledge and skills for all cadres of healthcare providers working in maternity wards in both sub-Saharan Africa and Asia. Additional support and training is needed for use of the partograph as a tool to monitor progress in labour. Further research is needed to assess if this is translated into improved service delivery.

## Introduction

Each year, an estimated 300,000 women worldwide die from complications of pregnancy and childbirth [1]. The time surrounding birth is considered the most critical period and requires effective care packages to be in place. Provision of skilled birth attendance and Emergency Obstetric Care (EmOC) for women and babies who require this are ‘bundles of care’ which, if in place, will reduce maternal and neonatal mortality and morbidity. A Skilled Birth Attendant (SBA) is "an accredited health professional–such as a midwife, doctor or nurse–who has been educated and trained to proficiency in the skills needed to manage normal (uncomplicated) pregnancies, childbirth and the immediate postnatal period, and in the identification, management and referral of complications in women and newborns" [2]. EmOC is defined by up to nine signal functions including ability to provide; intravenous or intramuscular oxytocic, antibiotic and anticonvulsant (preferably magnesium sulphate), manual removal of a retained placenta, manual vacuum aspiration of retained products of conception, ventouse delivery, resuscitation of the newborn using a bag and mask and caesarean section [3]. At least 80% of all maternal deaths result from five complications that are well understood and can be readily treated: haemorrhage, sepsis, eclampsia, and complications of obstructed labour and abortion [4]. Similarly, the majority of newborn deaths globally are the result of prematurity, birth asphyxia and sepsis [1]. Having the knowledge and skills to recognise and respond effectively in case such complications occur is a key part of a skilled attendant’s role.

In-depth assessments of availability and coverage of EmOC have shown that in many cases structures are in place, equipment and consumables available but staff are unable to provide all the signal functions of EmOC [5–8]. The combination of lack of knowledge and of skills is likely to be a key reason why many beneficial evidence-based practices are still not used in many resource poor settings [9]. EmOC training is included in up to 65% of all maternal and newborn health implementation programs in low and middle-income countries [10].

In this study our objective was to assess existing knowledge and skills of healthcare providers designated to provide SBA and EmOC across nine countries in sub-Saharan Africa and South East Asia. We assessed change in knowledge and skills after implementation of a standardised EmOC training package and explored factors associated with change in knowledge and skills for different cadres and settings and for specific components of the training including management of haemorrhage, sepsis, (pre-) eclampsia, obstructed labour, assisted vaginal delivery and newborn resuscitation.

## Methods

Between October 2012 and June 2014, healthcare providers in nine countries participated in EmOC training and were assessed before and after training (Bangladesh, Ghana, Kenya, Malawi, Nigeria, Pakistan, Sierra Leone, Tanzania and Zimbabwe). All cadres of healthcare providers providing maternity acre and considered to be skilled birth attendants could participate in the training. All groups trained were multi-disciplinary to reflect the ‘team’ of healthcare providers working on the maternity wards in each country.

### Training package

A standardised EmOC training package was developed in 2006 by the Centre for Maternal and Newborn Health at the Liverpool School of Tropical Medicine in partnership with the World Health Organization (WHO) and Royal College of Obstetricians and Gynaecologists (RCOG) [11]. The course is designed to include the essential knowledge and skills required by skilled birth attendants to recognise and manage the major causes of maternal and newborn death in low and middle income (LMIC) and includes all EmOC signal functions [3].

Specific topics covered during the training are: maternal and newborn resuscitation, early newborn care (recognition and management of prematurity, hypoglycaemia and hypothermia), communication triage and referral, management of shock and the unconscious patient, recognition and management of severe pre-eclampsia and eclampsia, recognition, prevention and management of obstetric haemorrhage, sepsis. use of the partograph, recognition and management of obstructed labour, ability to perform assisted vaginal delivery (ventouse delivery), manual removal of retained placenta and manual vacuum aspiration for retained products of conception, recognition and management of other obstetric emergencies (breech delivery, cord prolapse, twin delivery, shoulder dystocia) and managing difficult caesarean sections.

The training is delivered over three to five days (depending on country) using the same content and methodology for delivery in each country and applying a multidisciplinary approach with all cadres providing maternity care in any setting trained together by multidisciplinary teams of experienced facilitators who have participated in standardised ‘Training of Trainers’ (facilitator: participant ratio is 1:4–6).

The training is based on the key principles of adult learning and uses interactive learning sessions comprising of short lectures (15% of total time), simulation training using low fidelity obstetric, newborn and resuscitation mannequins (40%) role play and workshops (30%), mentoring (5%) and in-course monitoring and evaluation (10%).

### Assessment of knowledge and skills

The assessments were designed to test participants’ knowledge (all content of training) and skills (obstetric haemorrhage, pre-eclampsia, sepsis, manual vacuum aspiration, recognition of obstructed labour using a partograph, maternal and newborn resuscitation and assisted vaginal delivery). The protocol for testing was uniform across all countries; all participants were invited to take part in the assessment immediately before and after the training. Materials used in the two assessments were the same, giving a like-for-like comparison for each participant tested. Knowledge was assessed via 40 (true or false) questions randomly drawn from a bank of 240 questions and skills were assessed using four structured scenario-based assessments and four objective structured clinical examinations (OSCE).

Prior to the assessment, participants completed a self-administered anonymised questionnaire, which collected information on cadre, obstetric experience (in years), proportion of work-time spent on the labor ward/obstetrics (using an ordinal scale of 0%, 25%, 50%, 75% or 100%), midwifery or medical teaching role, and whether the participant had received in-service training in EmOC before.

### Analysis of data

Questionnaires were assigned unique identifying numbers to link before and after results for each participant. All answer sheets were marked, scanned (Formic computer program) and exported into SPSS version 24.0 for analysis. The total score that could be obtained was 320 (split evenly between knowledge and skills). Results are expressed as a percentage of total.

Cadre of healthcare provider was grouped into four groups based on duration and content of pre-service training 1) doctors; 2) mid-level staff; 3) nurses and midwives; and 4) nursing aides. Mid-level staff consisted of Clinical Officers (Malawi, Tanzania, Kenya); Assistant Medical Officers and Clinical Officers (Tanzania); Community Health Officers (Sierra Leone); and Medical Assistants (Malawi, Bangladesh). Nursing aides included all healthcare providers who conducted deliveries with a pre-service training that was shorter than nurses or nurse-midwives; Community Health Extension Workers (CHEW) (Nigeria), Mother and Child Health Aide (MCHA) (Sierra Leone).

Participants with a higher pre-training score have less potential for improvement compared to those with a low pre-training score. Additionally, there was more variability in absolute changes among those who performed less well pre-training. Therefore, to remove heteroscedasticity, we used an Improvement Ratio (IR) calculated by dividing the absolute improvement by the improvement potential (320 minus overall pre-training score) and converting this to a percentage (the proportion of maximum potential achieved).

For variation in knowledge and skills by area of care relevant to the leading causes of maternal and neonatal mortality, summary scores were calculated for: haemorrhage, sepsis, pre-eclampsia, obstructed labour, assisted vaginal delivery and newborn resuscitation. In these areas of care, an aggregate mean percentage score was calculated by giving equal weighting to relevant knowledge and skills for that component.

Step-wise linear regression was performed to identify the independent variables most associated with pre-training score and Improvement Ratio. Obstetric experience data was transformed into five categories as exploratory analysis showed a non-linear relationship with knowledge, skills and combined score (0–2, 3–5, 6–8, 9–11, >12 years) both pre-treatment and using the IR. The variables considered were country, cadre, obstetric experience in years, proportion of work in obstetrics, employed in midwifery/medical teaching job, and if they had received in-service obstetric/newborn skills training before. Categorical variables were converted to dummy variables and categories most frequently occurring used as reference categories. Scatter plots, histograms, tests of multicollinearity, and the Goldfeld-Quandt test were performed on the data to ensure it satisfied the assumptions required for linear regression. The dummy variables groups were entered into the step-wise linear regression in order of their R-square value (a, then a+b, then a+b+c, etc.). Coefficients are presented for the final model (a+b+c+d+e+f) as well as R-square values for the preceding models.

After tests of normality were performed, analysis of variance (ANOVA) was used to compare the mean scores and improvement ratios between cadres. The matched pairs signed rank test was used to test whether there was a significant improvement in median scores before and after training for all participants then sub-groups.

### Ethical considerations

Delivery and evaluation of the EmOC training was approved by the Ministry of Health in all countries. Completing assessments before and after the training was considered part of the learning experience but participants could opt out of the assessment if they so desired.

## Results

### Characteristics of participants

In total, 5,939 healthcare providers took part in the knowledge and skills testing before and after EmOC training (Table 1)(S1 File). Matched scores for before and after testing were available for knowledge (5,757 participants), skills (5,161 participants) and knowledge and skills combined (5,142 participants). The highest number of participants were from Kenya and the majority of participants were nurses or midwives. Over 80% of participants primarily provide maternity care. The majority (65.9%) had no more than five years of experience.

**Table 1:** Characteristics of healthcare providers trained in emergency obstetric and early newborn care

Characteristic	Number of Participants
	n (% of total)
**Country of origin**	
Bangladesh	538 (9.1)
Ghana	598 (10.1)
Kenya	1636 (27.5)
Malawi	206 (3.5)
Nigeria	526 (8.9)
Pakistan	373 (6.3)
Sierra Leone	472 (7.9)
Tanzania	699 (11.8)
Zimbabwe	891 (15.0)
**Cadre**	
Doctor/Medical Officer	867 (14.6)
Mid-level Staff	401 (6.8)
Nurse/Midwife	3045 (51.3)
Nursing aide	278 (4.7)
Other/missing	1348 (22.7)
**Proportion of work in maternity care/obstetrics**	
0%	307 (5.5)
25%	703 (12.7)
50%	1123 (20.2)
75%	1530 (27.5)
100%	1891 (34.0)
Missing	385 (6.5)
**Received previous in-service training**	
Yes	1594 (26.8)
**Midwifery or medical teaching job**	
Yes	376 (6.3)
**Number of years experience in maternity care/obstetrics**	
0–2	2082 (38.2)
3–5	1510 (27.7)
6–8	592(10.8)
9–11	462 (8.5)
12–44	811 (14.9)
Missing	482 (8.1)

### Knowledge and skills before and after training

Before training the participants’ median knowledge score was higher than the median skills score (70.0% vs 51.9%) (Table 2). Almost all participants (99.7%; 4,951 of 4,965) demonstrated an improvement in overall score following the EmOC training.

**Table 2:** Change in knowledge and skill after Emergency Obstetric and Newborn training for all participants, then combined score change for each cadre and country

	Pre-course median score (IQR)	Post-course median score (IQR)	Change in median score (IQR)
**All participants**			
Knowledge	70.0 (62.5–77.5)	80.0 (72.5–85.0)	10.0 (5.0–15.0)
Skill	51.9 (43.1–60.0)	82.5 (75.6–88.1)	28.8 (23.1–35.1)
Combined	61.3 (54.1–68.1)	81.6 (75.3–86.3)	19.4 (15.3–24.1)
**By cadre (knowledge and skills combined)**			
Doctor/ medical officer	71.3 (65.9–12.8)	87.5 (83.8–90.6)	16.6 (12.8–20.3)
Mid-level staff	59.7 (54.0–65.6)	80.0 (74.7–85.3)	20.0 (15.6–24.1)
Nurse/ midwife	60.0 (53.4–65.6)	80.3 (75.3–84.7)	20.0 (15.6–24.1)
Nursing aide	46.6 (39.6–50.9)	66.9 (60.9–71.8)	20.3 (15.3–25.6)
**By country of origin (knowledge and skills combined)**			
Bangladesh	68.4 (65.3–74.0)	84.7 (78.6–88.4)	16.6 (12.5–21.6)
Ghana	60.9 (55.6–66.9)	81.6 (76.6–86.3)	19.4 (15.6–24.0)
Kenya	61.3 (55.9–66.2)	82.8 (79.0–86.3)	20.9 (17.2–25.0)
Malawi	65.6 (60.9–69.8)	83.3 (80.1–87.2)	17.2 (13.4–21.0)
Nigeria	54.1 (46.0–60.3)	76.6 (68.4–82.2)	20.3 (15.9–25.6)
Pakistan	48.8 (40.7–59.0)	73.0 (64.4–80.6)	22.2 (16.9–29.1)
Sierra Leone	53.8 (45.9–60.3)	74.1 (68.4–80.0)	20.3 (14.7–25.0)
Tanzania	55.9 (50.3–62.5)	76.9 (71.3–81.6)	19.7 (15.3–24.1)
Zimbabwe	68.4 (64.1–72.8)	85.9 (81.9–89.0)	16.9 (13.8–20.6)

IQR = Interquartile Range

**Fig 1:**
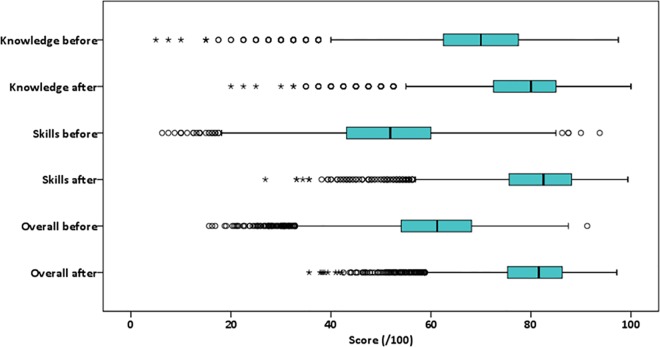
Box and whisker plots showing change in knowledge and skills after Emergency Obstetric and Newborn Care training

The median knowledge score (n = 5,757) improved from 70.0% before training to 80.0% after training. The median skills score (n = 5,608) improved from 51.9% to 82.5% (Fig 1). The greater median improvement in skills (28.8%) versus knowledge (10.0%) resulted in similar post training scores of around 80% for each component. The overall median score increased from 61.3% to 81.6% after training with a median improvement of 19.4%. There were statistically significant improvements in knowledge, skills and overall scores following training for each cadre group and in each country (p<0.05).

### Factors associated with pre-training score

Step-wise linear regression analyses found that the variables most strongly associated with pre-training scores were country and cadre which explained 37.9% of the variability in pre-training scores (Table 3). Adding in all available independent variables, the model explained 40.2% of the variability in pre-training score. Doctors and those in Zimbabwe scored the highest when compared to the reference categories, whereas those from Pakistan and nursing aides scored the least. Holding a teaching job, previous in-service training, and an increasing percentage of work- time spent in providing maternity care were each associated with a higher pre-training score. Those with more than 11 years of obstetric experience had the lowest scores prior to training, with mean scores 1.4% lower than those with no more than 2 years of experience.

**Table 3:** Step-wise linear regression for overall knowledge and skill score before training in emergency obstetrics and newborn care*.

Variable*	Step-wise model (a, a+b, a+b+c, …)	Final model (a+b+c+d+e+f)		
	R-Square	Coefficient in final model	std. error	p-value
(Constant)		57.70	0.41	<0.05
a. Country	0.243			<0.05
Bangladesh		-0.98	0.51	0.06
Ghana		-0.88	0.47	0.06
Malawi		4.08	0.87	<0.05
Nigeria		-6.79	0.54	<0.05
Pakistan		-13.13	0.57	<0.05
Sierra Leone		-6.12	0.52	<0.05
Tanzania		-4.92	0.42	<0.05
Zimbabwe		4.93	0.41	<0.05
b. Cadre	0.379			<0.05
Doctor/ medical officer		11.53	0.41	<0.05
Mid-level staff		2.06	0.52	<0.05
Nursing aide		-7.41	0.67	<0.05
Unknown		0.51	0.31	0.11
c. Years of obs. experience	0.387			<0.05
3–5 years		1.30	0.32	<0.05
6–8 years		1.12	0.43	<0.05
9–11 years		0.64	0.48	0.19
>11yrs		-1.42	0.40	<0.05
Unknown		-2.11	0.95	<0.05
d. Teaching job	0.393	3.72	0.55	<0.05
e. Training before	0.398	1.78	0.29	<0.05
f. Proportion of work in obs.	0.402	2.59	0.47	<0.05

* For country, cadre and years of experience estimates are contrasts with the reference categories: Kenya; Nurse/ Midwife; ≤2 years of experience.

### Factors associated with improvement in knowledge and skills

As with pre-training scores, improvement in knowledge and skills was most strongly associated with country and cadre. Together country and cadre explained 12.8% of the variability in improvement ratio (Table 4). Adding all known independent variables to the model explained 14.5% of the variability in improvement. Kenya scored the highest improvement ratios and Sierra Leone the lowest. Doctors had a mean improvement ratio which was 7.2% higher than for nurses, who themselves improved 6.5% more than nursing aides. Those with 3–5 years of experience achieved a similar improvement ratio to those with lesson more than 2 years of experience (0.6% higher). However, those with 9–11 years or more than 11 years of experience had smaller improvement ratios of 1.5% and 5.1% respectively. No significant association was found between improvement ratio and mid-level staff, having received training before, holding a teaching job, or between differing proportions of work in maternity.

**Table 4:** Step-wise linear regression for overall improvement ratio for knowledge and skill score after training in emergency obstetrics and newborn care

Variable*	Step-wise model (a, a+b, a+b+c, …)	Final model (a+b+c+d+e+f)		
	R-Square	Coefficient in final model	std. error	p-value
(Constant)		55.09	0.66	<0.05
a. Country	0.089			<0.05
Bangladesh		-8.17	0.83	<0.05
Ghana		-2.60	0.75	<0.05
Malawi		-4.52	1.39	<0.05
Nigeria		-8.33	0.88	<0.05
Pakistan		-9.26	0.92	<0.05
Sierra Leone		-9.59	0.84	<0.05
Tanzania		-8.62	0.67	<0.05
Zimbabwe		-2.05	0.66	<0.05
b. Cadre	0.128			<0.05
Doctor/Medical officer		7.21	0.67	<0.05
Mid-level staff		0.45	0.83	0.59
Nursing aide		-6.51	1.08	<0.05
Unknown		2.19	0.50	<0.05
c. Years of obs. experience	0.144			<0.05
3–5 years		0.65	0.51	0.20
6–8 years		-1.48	0.69	<0.05
9–11 years		-1.53	0.77	<0.05
>11yrs		-5.06	0.65	<0.05
Unknown		-3.70	1.51	0.02
d. Teaching job	0.145	1.54	0.90	0.09
e. Training before	0.145	0.30	0.47	0.52
f. Proportion of work in obs.	0.145	-1.09	0.75	0.15

* For country, cadre and years of experience estimates are contrasts with the reference categories: Kenya; Nurse/ Midwife; ≤2 years of experience.

### Comparing Countries

**Fig 2:**
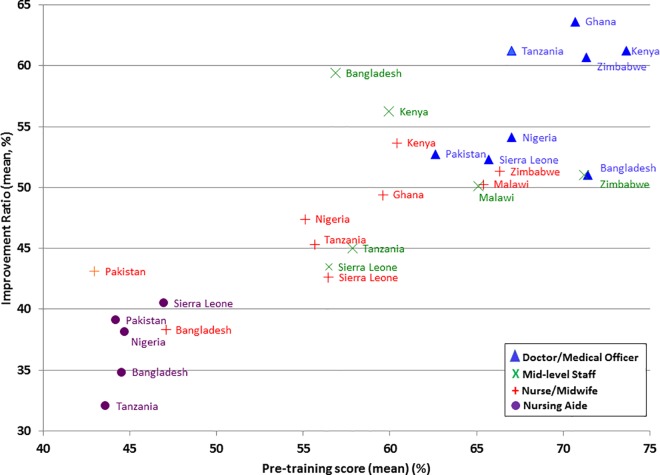
Mean improvement ratio and pre-training score by cadre and country

The mean pre-training score and improvement ratio were calculated for all cadre-groups in all countries (Fig 2). Healthcare providers in Ghana, Kenya, Zimbabwe and Malawi had higher mean pre-training scores and improved relatively more than healthcare providers from other countries. Doctors in Kenya obtained the highest pre-training scores (74%) and doctors in Ghana had the highest improvement ratio (64%). Among nurse-midwives, those from Zimbabwe and Malawi obtained the highest pre-training scores (both 66%).

Pakistan had the lowest mean pre-training scores for both doctors (63%) and nurses-midwives (43%). They also achieved comparatively lower improvement ratios (Pakistani doctors 51%, Pakistani nurse- midwives 43%). Bangladeshi doctors and nurse-midwives also obtained comparatively low scores prior to the training (71% and 47%, respectively). They showed the smallest improvement ratios for their cadres (Bangladeshi doctors: 39%, Bangladeshi nurse-midwives: 51%).

Sierra Leone’s doctors and nurse-midwives had mean pre-training scores of 66% and 57%, similar to other countries in sub-Saharan Africa (e.g. Nigerian doctors: 67%, Nigerian nurses/midwives: 55%). Despite this they achieved the smallest mean improvement ratios out of the seven African countries (Sierra Leonean doctors: 53%, nurses/midwives: 43%). Sierra Leone’s nursing aides, however, scored highest in their cadre in both pre-course score (47%) and improvement ratio (IR 41%).

### Cadre sub-analysis

Doctors scored significantly higher than all other cadres in every knowledge and skill assessment before training (ANOVA, p<0.05). Equally, nursing aides scored significantly lower than all other cadres in every pre-training assessment (ANOVA, p<0.05). Overall, mean pre-training score for doctors was 70%, mid-level staff 60%, nurse-midwives 59%, and nursing aides 45%. Overall, mean improvement ratios were: doctors 56%, mid-level staff 50%, nurse-midwives 50% and nursing aides 38%. There was no significant difference found between mid-level staff and nurse-midwives in either overall pre-training score or improvement ratio when tested with analysis of variance.

### Comparisons for areas of care included in training

**Fig 3:**
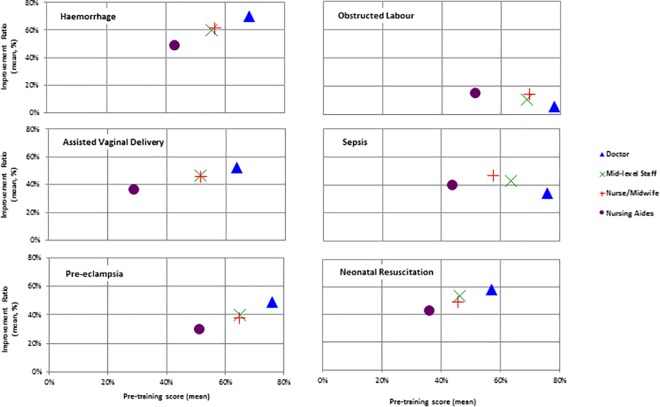
Mean improvement ratio and pre-training score by cadre for six main components of the “skills and drills” training package

For haemorrhage, assisted vaginal delivery, and (pre-) eclampsia the pattern of scores was similar to that obtained overall. Doctors scored the highest and improved the most; nursing aides scored the lowest and improved the least. In these modules nurse-midwives did not perform statistically significantly differently from mid-level staff with regard to either pre-training score or improvement ratios. (Fig 3) The greatest improvement ratio in all cadres was for haemorrhage (49–70%). For obstructed labour all cadres achieved their highest score pre-training (51–78%) but improved the least (6–15%). For sepsis, mid-level staff performed better than nurse-midwives before the course (63% vs 58%, ANOVA p<0.05) but nurse-midwives improved the most, with a higher improvement ratio than doctors (47% vs 34%, ANOVA p<0.01).

In neonatal resuscitation, nurses-midwives and mid-level staff both scored a mean of 46% before the training, but nursing staff improved significantly more with a mean improvement ratio of 54% (versus 49%, ANOVA p<0.01). Neonatal resuscitation was the weakest area pre-training for all cadres except nursing aides, who had assisted vaginal delivery as their weakest area.

## Discussion

### Main findings

This study shows that across nine countries in sub-Saharan Africa and south East Asia, in each country setting and for each cadre of healthcare provider, there was a significant improvement in knowledge and skills after receiving a short competency-based training package in emergency obstetrics and early newborn care. Healthcare providers who had been in post for more than 10 years were less able to improve their scores compared to those who had completed training more recently and been in post for a shorter period of time. However, the proportion of work-time spent providing maternity care was not associated with an improvement in scores. Healthcare providers with shorter pre-service training (nursing aides) and nurse-midwives from Bangladesh and Pakistan had the lowest pre-training scores and demonstrated the least improvement in key areas of emergency obstetric and early newborn care. Knowledge and skills in recognition and management of obstructed labour using the partograph were least improved following training and this was highest overall for recognition and management of obstetric haemorrhage.

### Strengths and limitations

Over half of all intervention programs aimed at reducing maternal and newborn mortality and morbidity in low- and middle-income countries include a component of training in emergency obstetrics and/or newborn care. However, there is limited evidence to show the effect of this. To the best of our knowledge, this is the largest study evaluating whether there is improvement in knowledge and skills after such training and includes all the relevant cadres of healthcare providers working in the maternity areas of healthcare facilities from nine different low and middle income countries. In addition, we related change in knowledge and skills to potential for improvement using an improvement ratio rather than using only absolute change in scores.

The study was conducted under the Making it Happen program for which target districts in each country have relatively poor maternal and newborn health indicators compared to the national average. All healthcare providers working in the maternity area of government or public healthcare facilities designated to provide emergency obstetric care participated in the training. Therefore, the selection of participants included in this study is pragmatic but representative of the workforce in place in these settings. The training was well defined with standardised delivery possible across all settings and we conducted multi-modality testing under exam conditions. Our study assessed the change in knowledge and skills following ‘skills and drills’ type in-service training and the factors associated with this change, using a pragmatic before-after study design. Although we recognise that simultaneous testing of ‘controls’ (i.e. healthcare providers not offered training) might have demonstrated a stronger cause-effect relationship, this was not considered to be justified and would have been difficult to implement in practice. Similarly, our study population was heterogeneous to reflect the day-to-day mix of different cadres of healthcare providers who provide maternity services in each country setting. Cadre and country were most strongly associated with improvement ratio, but these only explained 12.8% of the variability across the 11 countries.

### Interpretation

A number of training packages have been evaluated at healthcare provider level, specifically assessing reaction to the training and change in knowledge and skills after training. Most of these have reported improvement in knowledge and skills but all used absolute change in scores rather than the mean improvement ratio (which accounts for maximum potential to improve and variability between participants) [12–20]. Training packages have generally not been well defined with regard to content and methodology of delivery and therefore comparison across multiple, smaller studies from different settings has been difficult [12,21]. It has also not been possible to assess learning across the different components of a training package. Factors associated with pre-training scores or change in knowledge and skills observed in various settings have not been previously described. We studied variables that could be expected to be associated with change in knowledge and skills after training or with level of pre-existing knowledge and skills. These included proportion of time spent working in the maternity area and number of years of work experience. Although more recently qualified healthcare providers had both a lower pre-training score and larger improvement ratio, this difference was not large. Improvement was most strongly associated with country and cadre.

Overall, healthcare providers in Asia had both lower pre-training scores and improvement ratio compared to similar cadres of healthcare providers from sub-Saharan Africa. This is particularly noticeable among nurse-midwife cadres, where the test results show the greatest discrepancy of pre and post training scores between countries. [22,23]. In addition, even where the total length of pre-service training is similar, the content with regard to obstetrics and related services is often different. However, we were not able to assess content of pre-service training in this study and this requires further research. In Asia, there are in general more specialist obstetrician-gynaecologists in post who oversee and support the work of nurse-midwives who therefore may have less experience providing emergency obstetric care. Finally, we note that in many south-East Asian countries nurse-midwifery training is not conducted in the English language. The training workshops as well as assessments used in the programme were, however, in English. It may be important to provide more of the training and assessment materials in other languages.

In this study knowledge and skills were equally ‘weighted’ as were the different components of the training assessed. This may be incorrect as not all components of obstetric and newborn care lend themselves equally well to ‘skills and drills’ type training and overall the emphasis is on skills rather than knowledge improvement. This might explain why the greatest effect was seen with regard to recognition and management of obstetric haemorrhage which lends itself to simulation-based or ‘skills and drills’ type training. In contrast, despite high pre-training scores for use of the partograph there was comparatively little improvement after training and this might require a different training approach.

## Conclusions

Sub-optimal care in many cases contributes to maternal and neonatal deaths and this includes inability of healthcare providers to recognise and manage complications of pregnancy and childbirth in a timely and effective manner. Evaluation of effectiveness of training is difficult when this is part of a wider implementation programme with multiple interventions. Secondly, in most cases the interventions including EmOC training packages have been poorly described with regard to content and method of delivery making attribution difficult.

The theory of change and model for evaluation of training of healthcare providers would suggest that improving knowledge and skills is a pre-requisite to catalysing change in behaviour and clinical practice. This can be challenging in resource poor settings where the enabling environment is not in place and further research is needed to assess if this does occur and how [5,22]. Change in behavior and clinical practice is expected to contribute to improved availability and quality of care and a reduction in number of women and babies with complications (as these may have been prevented) and a reduction in case fatality rates for women and babies with complications when these do occur [24,25].

## Supporting Information

S1 File Data File: Knowledge and Skills Testing Results (SAV)
